# Uncovering root compaction response mechanisms: new insights and opportunities

**DOI:** 10.1093/jxb/erad389

**Published:** 2023-11-09

**Authors:** Bipin K Pandey, Malcolm J Bennett

**Affiliations:** Plant and Crop Science, School of Biosciences, University of Nottingham, Sutton Bonington Campus, Leicestershire LE12 5RD, UK; Plant and Crop Science, School of Biosciences, University of Nottingham, Sutton Bonington Campus, Leicestershire LE12 5RD, UK; CSIRO Agriculture and Food, Australia

**Keywords:** ABA, auxin, ethylene, root responses, soil compaction

## Abstract

Compaction disrupts soil structure, reducing root growth, nutrient and water uptake, gas exchange, and microbial growth. Root growth inhibition by soil compaction was originally thought to reflect the impact of mechanical impedance and reduced water availability. However, using a novel gas diffusion-based mechanism employing the hormone ethylene, recent research has revealed that plant roots sense soil compaction. Non-compacted soil features highly interconnected pore spaces that facilitate diffusion of gases such as ethylene which are released by root tips. In contrast, soil compaction stress disrupts the pore network, causing ethylene to accumulate around root tips and trigger growth arrest. Genetically disrupting ethylene signalling causes roots to become much less sensitive to compaction stress. Such new understanding about the molecular sensing mechanism and emerging root anatomical traits provides novel opportunities to develop crops resistant to soil compaction by targeting key genes and their signalling pathways. This expert view discusses these recent advances and the molecular mechanisms associated with root–soil compaction responses.

## Teaser

New insights into novel molecular and cellular mechanisms that control soil compaction responses in model plant roots provide translational opportunities to select more resilient crops.

## Introduction

**Fig. 1.**
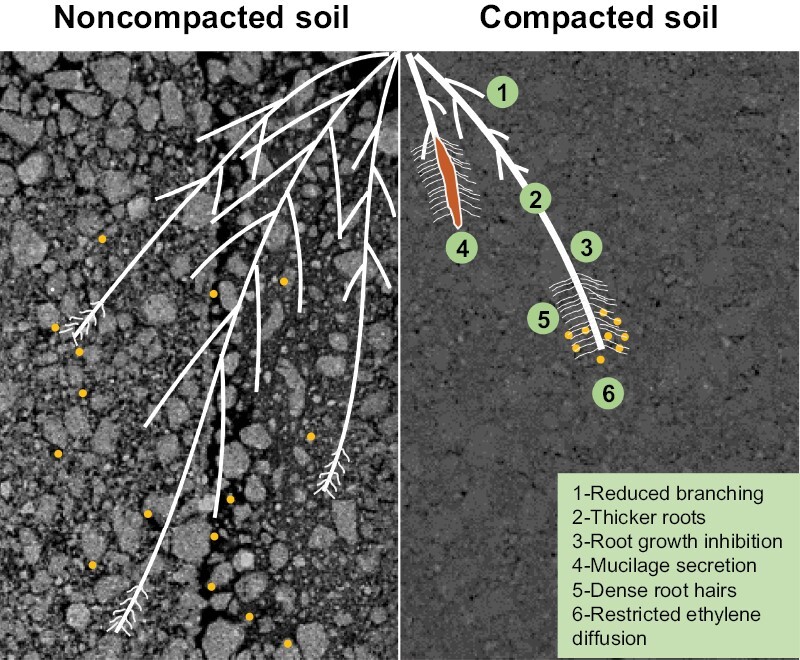
Root responses in compacted soil conditions. The left side of the image illustrates root soil compaction responses in non-compacted soil conditions that allow for the optimal diffusion of ethylene through connected soil pores, resulting in favourable root growth responses. In contrast, the right side of the image depicts the effects of soil compaction (reduced soil pore volumes), which restricts the diffusion of ethylene, causing a reduction in root growth. Compaction has several effects on root soil responses, including the secretion of mucilage, reduced branching, thicker roots, dense root hairs, and decreased water infiltration and gaseous exchange. Ethylene is represented by filled yellow circles.

Soil compaction has been a longstanding challenge in agriculture but has been regarded as a controllable problem through effective management practices (Batey, 2009). However, the widespread adoption of heavier machinery in the agricultural sector has transformed soil compaction into an inescapable risk, which presents formidable obstacles to modern farming practices (Håkansson and Reeder, 1994). Compaction not only reduces root growth and limits access to critical resources in deeper soil layers, but it also has a significant impact on the water infiltration, gaseous exchange, microbial activity, and water retention capacity of the soil (Fig. 1) (Nawaz *et al.*, 2013). When combined with other soil stresses such as drought, the yield loss in compacted soil ranges from 20% to 75% depending on soil texture and severity of stress (Correa *et al.*, 2019). Europe has 33 Mha of soil prone to compaction (Nawaz *et al.*, 2013), while compaction affects around 4 Mha of farmlands in England and Wales. Recent studies have revealed new insights into how plant roots sense soil compaction, as well as novel strategies to generate compaction-resistant genotypes (Pandey *et al.*, 2021; Schnieder *et al.*, 2021; Vanhees *et al.*, 2022; Huang *et al.*, 2022). This review presents recent key developments, current understanding, and future directions relating to root responses to compacted soil and selecting more resilient crops.

## Modern farming practices risk the rooting ability of crop plants

Modern agricultural practices rely heavily on mechanization, which can lead to critical subsoil compaction that is challenging to overcome using current management practices. While topsoil compaction (below 20–30 cm of tilled layers) can be alleviated using improved traffic management and tillage practices, subsoil compaction (below 50 cm) can create a hard soil layer, known as hard pan, that inhibits the rooting ability of crops (Berisso *et al.*, 2012). Since the green revolution, the weight of farming equipment has increased >10-fold, with some machines now having a footprint size (8800 cm^2^) as large as the heaviest dinosaurs (7000 cm^2^) on Earth (Box 1) (Keller and Or, 2022). Unfortunately, this trend towards larger, heavier equipment is exacerbating the problem of subsoil compaction, and it is becoming increasingly challenging to manage its effects on crop growth and yields.

## Down to earth problems: the complexities of soil compaction

Soil is a highly heterogeneous growth medium for plant roots. A well-structured soil is primarily composed of soil aggregates (~50%), water (~25%), and air-filled pores (~25%). When compacted, this ratio changes significantly to contain a higher proportion of soil particles and diminished air spaces and water content (Gupta *et al.*, 1989). This has a profound impact on the biophysical interaction between roots and soil, causing enhanced mechanical impedance, restricted gas exchange, and reduced water availability. As a result, root tip growth is severely reduced when encountering compacted soil conditions (Unger and Kaspar, 1994).

In a well-structured soil, macropores are highly interconnected, facilitating gas exchange. Soil compaction presses soil aggregates and breaks the connectivity between soil macropores, resulting in a reduction in macropore volume. Our recent findings suggest that plant roots use the gaseous hormone ethylene to indirectly sense soil compaction. Ethylene is released by root tips, and normally rapidly diffuses away in non-compacted soil, but gets trapped close to root tips exposed to compacted soil conditions (Fig. 1). Its accumulation triggers a root ethylene response, that acts as a growth stop signal (Box 1) (Pandey *et al.*, 2021). Unlike the wild type, roots of ethylene-insensitive plant mutants can penetrate highly compacted soil as they cannot detect the accumulation of ethylene. This surprising discovery suggests that wild-type roots stop growing in compacted soil due to ethylene accumulation rather than when physically forced by mechanical impedance, which runs counter to the established thinking in this field.

## Root adaptive responses in compacted soil

### Root growth inhibition

Soil compaction reduces root tip growth by limiting epidermal cell elongation (Pandey *et al.*, 2021) (Box 1). Epidermal cells make up the outermost tissue encapsulating the other root tissues, and play a key role in enabling the root to expand in both longitudinal and radial directions. A recent study reported that higher auxin accumulation in epidermal cells inhibits their axial expansion in the root elongation zone (Huang *et al.*, 2022) (Box 1). Interestingly, coarse root length (in non-compacted soil) does not correlate with root penetration depth in compacted soil in several maize genotypes (Vanhees *et al.*, 2021).

### Root radial expansion

Increased width of root tips is a hallmark adaptive response in compacted soil. This compaction response is conserved across a wide range of plant species including Arabidopsis, rice, tomato, barley, maize, wheat, and rye (Materechera *et al.*, 1992; Pandey *et al.*, 2021; Huang *et al.*, 2022). Earlier studies suggested that the radial expansion of roots is advantageous in compacted soil as thicker roots have greater resistance to penetration than thinner roots, thereby avoiding root buckling (Whiteley and Dexter, 1982; Clark *et al.*, 2003; Chimungu *et al.*, 2015). However, recent evidence revealed that roots that exhibit less radial expansion can penetrate better than roots that exhibit a higher swelling response in compacted soil conditions (Pandey *et al.*, 2021; Vanhees *et al.*, 2022; Huang *et al.*, 2022). Moreover, Vanhees *et al.* (2021) also found that the root thickening response of maize genotypes has no advantage for root penetration compared with genotypes whose roots remain thin. Huang *et al.* (2022) recently reported that abscisic acid (ABA) regulates compaction-induced root radial expansion. Although there is no known direct relationship between ethylene and regulation of ABA biosynthesis, ethylene does induce ABA biosynthesis by promoting expression of ABA biosynthesis genes such as *MHZ4/ABA4* (Ma *et al.*, 2014).

### Root hairs

Root hairs provide anchorage during seedling establishment (Bengough *et al.*, 2016). However, their role in aiding root penetration through compacted soil is unclear (Goss and Russel, 1980; Haling *et al.*, 2013). Nonetheless, recent research suggests that root hairs may affect the mechanical properties of the rhizosphere, including reducing soil hardness and elasticity (Marin *et al.*, 2022). One major challenge is in imaging microscopic root hair length and density in compacted soil (Fig. 1). This will require X-ray microscopy levels of resolution available using a synchrotron facility (<1 µm) which is not possible with conventional micro-computed tomography (CT; >5 µm) and MRI imaging systems.

### Root tip shape

The shape of the root tip appears to impact root penetration ability. Colombi *et al.* (2017); Box 1) reported in wheat that varieties better able to penetrate compacted soil featured a ‘sharper’ root tip shape, whereas varieties with a ‘rounder’ root tip shape struggled to penetrate hard soil profiles. Tip shape appears to be controlled by ethylene as insensitive mutant roots remain ‘sharp’ rather than becoming ‘flattened’ by exposure to compacted soil (Pandey *et al.*, 2021).

### Mucilage secretion

Soil compaction induces root tip mucilage secretion, presumably to reduce the frictional force between soil particles and root cap cells (Iijima *et al.*, 2000). Root mucilage is made up of complex carbohydrates, which help tips to elongate in compacted soil (Oleghe *et al.*, 2017). Moreover, mucilage can increase the hydraulic conductivity and water uptake in rhizosphere soil (Carminati and Vetterlein, 2013). Thus, inducing mucilage secretion in compacted soil can help enhance root hydraulic conductivity, thereby countering the reduction in compacted soil water release properties.

Box 1. **Key discoveries underpinning root responses to soil compaction**

**(A) Chronic subsoil compaction is caused my modern dinosaurs.**

Keller and Or (2022) reported that heavy machinery, such as combine harvesters (weighing up to 36 Mt), has exceeded the critical mechanical limits (wheel load >5 Mt) and is responsible for chronic subsoil compaction affecting the highly sensitive crop root zone (below 50 cm). Their data analysis revealed that the pre-compression stress induced by these combine harvesters is much higher in dry topsoil than in wet topsoil (0–50 cm), while pre-compression stress is less in deeper soils (beyond 50 cm). This simply means that deeper soil layers are more susceptible to compaction (subsoil) in comparison with the topsoil region.

**(B) Ethylene diffusion defines how plant roots sense soil compaction.**

Pandey *et al.* (2021) discovered that roots use the gaseous hormone ethylene to sense soil compaction due to changes in their gas diffusion properties. Ethylene accumulation around the root tip inhibits root elongation and promotes radial expansion. The researchers also reported that mutants no longer able to sense ethylene can penetrate compacted soil better than the wild type. Hence, ethylene (rather than mechanical impedance) triggers root growth arrest.

**(C) ABA and auxin regulate root responses in compacted soil.**

Huang *et al.* (2022) described how ethylene modulates two distinct hormone signals, ABA and auxin, to regulate the radial expansion of root cortical cells and epidermal cell expansion, respectively, in compacted soil conditions. The study showed that ABA biosynthesis genes are induced in compacted soil conditions, leading to higher ABA levels and causing swelling in root tip tissues. Additionally, the research explains that higher auxin accumulation in epidermal cells restricts expansion, leading to reduced root growth in compacted soil conditions.

**(D) MCS provides mechanical strength for root penetration in compacted soil.**

Schneider *et al.* (2021) showed that highly lignified outer cortical cells, called multiple cortical sclerenchymatous (MCS) cells, provide mechanical stability in maize roots by reducing buckling, helping them penetrate compacted soil.

**(E) Tip shape impacts root penetration ability.**

Colombi *et al.* (2017) reported in wheat that varieties better able to penetrate compacted soil featured a ‘sharper’ root tip shape, whereas varieties with a ‘rounder’ root tip shape struggled to penetrate hard soil profiles.

### MCS formation

Another strategy that plants employ to overcome compacted soil conditions is through the formation of multiseriate cortical sclerenchyma (MCS). This lignified outer cortical cell is present in most cereal crop species, including maize, wheat, and barley (Schneider *et al.*, 2021; Box 1). It is reported to provide mechanical stability, allowing root tissues to penetrate compacted soil more effectively. This lignified outer root layer is also likely to aid water conservation in roots growing through hard soils with low water release properties. Interestingly, MCS formation is induced by the key compaction regulatory signal, ethylene.

### Branching

Root exploration of soil is vital for plants to secure key resources such as water and nutrients, and lateral root branching is a major contributor to this. Soil compaction has a significant impact on all root classes including lateral roots. In roots exposed to compacted soil conditions, branching density is severely reduced (Colombi *et al.*, 2017). Moreover, compacted soil slows down branching initiation in several crop species including wheat, soybean, and tomato (Tracy *et al.*, 2012; Colombi and Walter, 2015, 2017). While the mechanism behind this is not entirely clear, less water availability in compacted soil conditions could be a key factor for reduced branching density (Box 2). Interestingly, this reduction in lateral root branching mimics the xerobranching responses (Mehra *et al.*, 2022) which, like selected compaction responses, are regulated by ABA.

Box 2. **Open questions for future research in soil compaction**

1- How does root tip swelling help root penetration in compacted soil?
2- In what ways do soil compaction and soil drying stresses intersect to influence the molecular responses in compacted soil?
3- How do textural differences and moisture heterogeneity in macro-, meso-, and micropores impact gaseous exchange in compacted soil.
4- Which root plastic responses facilitate nutrient and water foraging in compacted soil?
5- How does compaction alter the microbiome community in the rhizosphere?

## Concluding remarks and future perspectives

Understanding how to mitigate the negative impact of compaction is of paramount importance for developing agronomic solutions. Several management strategies have been developed, including the use of controlled traffic farming (CTF), reducing tyre inflation pressure, using smaller farming vehicles, and adopting minimum or no tillage practices. Developing deep rooting, compaction-resistant crop varieties provides an alternative plant breeding-based solution. We discuss the key scientific questions that need to be addressed before a crop breeding-based approach can be delivered and also consider other plant-based opportunities to deliver agronomic solutions for compaction stress.

### A silver bullet solution: can ethylene resistance be used as a proxy to select compaction-resistant crops?

Our studies in rice have shown promising results, indicating that it may be possible to mitigate the negative effects of soil compaction on root growth by manipulating ethylene response (Pandey *et al.*, 2021). In addition to our findings, research conducted by Vanhees *et al.* (2022) on maize roots also suggests that disrupting ethylene response can alleviate the negative effects of soil compaction.

To further explore the potential of this approach, screening crop diversity collections for variation in ethylene sensitivity and then testing their compaction sensitivity could be a promising strategy to undertake in the near future. By identifying crop varieties that are less sensitive to soil compaction through ethylene response manipulation, we may be able to develop more resilient and productive crops that can better withstand compacted soil conditions.

### Does the ethylene gas diffusion mechanism extend to a range of compacted soil types?

To date, this mechanism has only been tested in a limited number of soil types—sandy loam and clay loam (Pandey *et al.*, 2021). A wider range of soil types and textures are required to be tested to predict (with confidence) whether this is a universal mechanism or to reveal that it is soil type/texture dependent. Different soil textures have varying effects on soil pore sizes during compaction stress, which will affect water and gas exchange. Therefore, direct measurement of gas exchange and imaging of water at the pore scale are needed, which will require use of sophisticated gas measuring tools in combination with high-resolution CT, MRI, and/or neutron tomography. By exploiting these tools, we can improve our understanding about how soil compaction affects the movement of gases and water in soils, which is essential for managing soil systems and optimizing agricultural productivity.

### Compaction versus drought: unlocking common molecular mechanisms

Both soil compaction and drought stress make soils harder and stronger, which can be difficult for root tips to penetrate. Given these apparent similarities, do the molecular mechanisms underlying how plants sense and respond to water stress and mechanical impedance overlap? Both adaptive responses employ common signals such as ABA (Huang *et al.*, 2022; Mehra *et al.*, 2022). To uncover common molecular mechanisms, advances in spatial transcriptomics provide new opportunities to reveal where/when/which genes are differentially expressed and identify common mechanistic signatures. Single-cell RNA sequencing represents a particularly promising technique to reveal common mechanisms. However, existing studies have been limited to profiling cellular expression for roots grown in agar, rather than soil, to date. This approach can help to elucidate the molecular and physiological responses of roots to compaction and drought stress directly in soil, which can inform the development of new strategies to mitigate the negative impacts of these stressors on plant growth and productivity.

### Under pressure: the science behind root swelling in compacted soil

The use of cover crops such as field radish to punch through compacted soil layers and create bio-pores is a well-established agronomic practice, but how do cover crops achieve this? Are their roots highly resistant to ethylene, enabling them to penetrate compacted plough pans and subsoil profiles? In the case of field radish, does the later secondary thickening of the root facilitate the formation and enlargement of biopores?

### Microbial makeover: peeking into the compacted underground

Soil compaction reduces soil pore spaces, which can accentuate resources (such as nutrients and water) for microbial growth. However, it is unclear how soil compaction drives the enrichment of specific microbes and repression of others. To understand microbiome dynamics in compacted soil, new imaging technologies such as SEERFISH in combination with high-resolution CT or MRI can be integrated to image microbial communities in compacted soil micropores. This approach can help reveal how soil compaction affects microbiome composition and function and provide insights into their potential for improving soil properties.

In conclusion, understanding the impact of soil compaction on plant growth and ecosystem services requires advanced techniques such as genomic resources, imaging facilities, and high-resolution physiological approaches. These approaches can help to elucidate the molecular and physiological responses of roots to soil compaction and reveal gas and water movement in soils. By identifying the key signals and mechanisms that affect plant growth in compacted soil, we can develop effective strategies to optimize agricultural productivity and mitigate the negative impacts of soil compaction on crop performance. Future research should focus on integrating these advanced techniques to further advance our understanding of soil compaction to mitigate its impact on crop performance and yield.
